# The therapeutic effect of repetitive transcranial magnetic stimulation in elderly depression patients

**DOI:** 10.1097/MD.0000000000021493

**Published:** 2020-08-07

**Authors:** Lilei Dai, Peng Wang, Panpan Zhang, Qingshan Guo, Hui Du, Fen Li, Xinfu He, Rongrong Luan

**Affiliations:** a Department of Clinical Psychology, Jingmen NO.2 People's Hospital, Jingmen, Hubei; b Department of Psychiatry, The Affiliated Xi’an Central Hospital of Xi’an Jiaotong University, Xi’an, Shaanxi; c Department of Clinical Psychology, Jingmen Oral Hospital, Jingmen, Hubei, China.

**Keywords:** depression, elderly patients, repetitive transcranial magnetic stimulation, suicidal ideation

## Abstract

**Background:**

Depression, a common psychiatric disorder in elderly, serves as a remarkable precipitating factor for suicide among the elderly people. Here, a randomized double-blinded study was performed to investigate the efficacy of repetitive transcranial magnetic stimulation (rTMS) on improving the clinical symptoms and reducing suicidal ideation in elderly patients with depression.

**Methods:**

In this study, 103 elderly patients with depression and suicidal ideation were randomly divided into 2 groups, 48 cases in the rTMS group and 55 cases in the control group (sham rTMS). Both groups received routine drug therapy with rTMS or sham rTMS. The patients received evaluation by Hamilton depression scale and self-rating idea of suicide scale before treatment and after 2 and 4 weeks of treatment, respectively.

**Results:**

The measurement from the present study demonstrated that Hamilton depression scale and self-rating idea of suicide scale scores decreased to varying degrees in the 2 groups after treatment, and the decrease was more significant in rTMS group. The rate of marked effectiveness was much higher in rTMS group after 2 weeks of treatment compared with the control group. Furthermore, the rate of moderate effectiveness at 4 weeks after treatment was significantly higher in rTMS group compared with the control group.

**Conclusion:**

Together, the present study shows that rTMS with routine drug therapy exhibited effect with quick onset to improve the clinical symptoms and reduce suicidal ideation in elderly patients with depression.

## 1 Introduction

It is estimated by the World Health Organization that the incidence of depression among the elderly people is 3% to 10% worldwide. Elderly depression is featured as persistent depressive mood after age of 60.^[1]^ Depression is a precipitating factor for suicide among the elderly people,^[2]^ and about 60% of the elderly patients with depression are at the risk of suicide.^[1,3]^ Drug therapy is the preferred option for depression and suicidal ideation, while the risk of suicide persists for the patients who take antidepressants until recovery.^[3]^ Elderly patients usually have the comorbidity of somatic illness^[4,5]^ and are more vulnerable to adverse effects of antidepressants,^[6]^ which substantially reduces the drug options and initial dose of antidepressants. This leads to slow or poor effect and further jeopardizes the prognosis and patient compliance in elderly patients.^[7]^

Repetitive transcranial magnetic stimulation (rTMS) is a new neurophysiological technique with extensive applications in neuropsychiatric field.^[8,9]^ It is well documented that rTMS is a noninvasive approach of brain stimulation to deliver the artificial magnetic field, which exhibits remarkable function to regulate the neuronal activity and synaptic plasticity in brain through electromagnetic induction.^[10,11]^ The potential effects of rTMS on the neuronal and brain function generally depend on the various parameters of stimulation, including waveform, frequency, intensity, and duration of stimulation. Previous studies showed that rTMS effectively reduced suicidal risk for young and middle-aged adults with depression.^[12,13]^ Recent studies also reported that rTMS with drug therapy exhibited quick onset of effects, shortened the course of treatment, and reduced suicidal ideation in young and middle-aged adult patients with depression.^[11,14]^ Currently, accumulating evidences existed to demonstrate the potential effectiveness of rTMS to treat the depression in adolescent and adult patients, while the efficacy of rTMS in elderly patients with depression is not reported yet.

Currently, a major portion of depression patients, especially the elderly, did not respond optimistically to the routine antidepressant therapy, which suggested an essential clinical need for additional antidepressant treatments.^[15,16]^ The present study focused on the therapeutic effect of rTMS in elderly patients with depression, especially its effect to reduce suicidal risk, which provided additional evidence for the clinical utilization of rTMS as an effective treatment for elderly patients with depression. In this article, active or sham rTMS, together routine drug therapy, was applied in the different cohorts of elderly patients with depression, who received evaluation by Hamilton depression scale (HAMD) and self-rating idea of suicide scale (SIOSS) before treatment and after 2 and 4 weeks of treatment, respectively. The purpose of this study was to define the potential therapeutic effect of rTMS to improve the clinical symptoms and reduce the suicidal ideation in elderly patients with depression.

## 2 Participants and methods

### 2.1 Participants

This is a randomized, double-blind, parallel-group design study. This study was approved by the ethics committee in Jingmen NO.2 People's Hospital (2015-018). Informed consent was obtained from each patient and the relatives. All cases were inpatients at the Department of Clinical Psychology of Jingmen NO.2 People's Hospital from April 2015 to April 2018.

### 2.2 Inclusion and exclusion criteria

The inclusion criteria include as following:

(1) They were diagnosed according to ICD-10 Criteria for Diagnosis of Depression;
(2) The diagnosis was confirmed by 1 attending physician and 1 associate consultant in the Psychiatry department. The cases presented with typical clinical symptoms of depression, with HAMD score ≥20 and SIOSS score ≥12;
(3) The cases, males or females, were aged at 60 to 80 years old;
(4) They had not taken any antidepressants within 3 months.

In general, HAMD score is the most often used tool for clinician-administered depression assessment, which extensively evaluate the psychiatric status of patients based on 17-item scale, such as depressed mood, feelings of guilt, suicide, insomnia, work and interests, retardation, agitation, psychiatric anxiety, and others. SIOSS is one of the widely used questionnaire to evaluate the suicidal ideation, in which it includes despair factor, optimistic factor, sleep factor, and masking factor, and the total score was calculated as the sum of these 4 factors.

The cases were excluded if any of the following criteria was met:

(1) history of severe somatic disease and organic disease of brain;
(2) depression caused by psychoactive substances or nondependent substance abuse;
(3) psychogenic depression;
(4) poor communication ability that made the evaluation difficult;
(5) carrying metallic materials in the brain or pacemaker or stent in the heart, having received crystalloid fluid replacement for cataract, having a personal or family history of epilepsy, or during gestation or lactation period;
(6) dropouts;
(7) high risk of suicide which need treatments in a closed environment.

### 2.3 Randomization and masking

The patients were randomized into active rTMS or sham rTMS group with simple randomization. Sample size in each group was all consistent with the previous studies. The patients and the evaluators were all blinded to the grouping and treatment. The investigators (Dr Luan) who generated the random allocation sequence were blinded to those who enrolled participants and those who assigned participants to interventions.

### 2.4 Measurements

General information including age, gender, ethnic group, education, occupation and, marital status was collected. The 24-item version of HAMD was used to evaluate the severity of depression, which consisted of 7 factors (anxiety/somatization, body mass, cognitive disorder, diurnal mood variation, sleep disorder, and sense of despair). SIOSS consists of 4 factors (despair factor, optimistic factor, sleep disorder, and mask factor) with 26 items, and the higher SOISS score means a stronger suicidal ideation. No change of measurement occurred after the study commenced.

### 2.5 rTMS

TMS device was manufactured by Magstim (United Kingdom). Figure of 8 coil and following treatment parameters were used based on preliminary experiments: stimulation frequency at 10 Hz, intensity of 100% motion threshold (MT, the minimum intensity of magnetic output to induce motor evoked potential), duration of 20 minutes. rTMS was applied at left prefrontal lobe for 4 seconds per minute with an interval of 56 seconds (ie, 800 pulses per day) in 5 times per week for 4 weeks.

### 2.6 Study procedure

Before treatment, all cases received psychological tests using HAMD and SIOSS, and the severity of depression and suicidal ideation were assessed. Cases with HAMD ≥20 and SIOSS ≥12 were randomly divided into experimental group and control group, both of which received routine drug therapy (escitalopram oxalate with initial dose 5 mg/d, which was adjusted based on patients’ condition 3 days later and gradually increased to 10 mg/d, with the maximum dose ≤20 mg/d). Escitalopram oxalate is a selective serotonin reuptake inhibitor by binding to serotonin transporter, and consequently inhibits serotonin reuptake and increases the amount of serotonin in synaptic clefts, by which resulting in antidepressant action. To ensure the safety and health of participants, no placebo group (without any drug treatment) was included as antidepressant therapy is currently available to effectively treat the patients with depression. For the experimental group, rTMS, starting at the first day of drug therapy, was given with a frequency of 5 times per week for 4 weeks. Pseudo stimulation was given in control group with the stimulation coil rotated by 90° in horizontal plane (tangential to skull), in which the field of magnetic stimuli was out of the skull. The cases were assessed again using HAMD and SIOSS at 2 and 4 weeks after treatment. All personnel were repeatedly trained for 3 days before treatment to be fully informed of the objective, requirement, methodology, and content of the study, so that a high consistency could be achieved between the personnel. The patients were informed the procedure and potential adverse effects of rTMS. During the process, the patients and raters were all blind to the treatment in individual case.

All cases were closely observed by attending physicians, nurses, therapists, and psychological assessors. The relatives were immediately informed if suicidal ideation was aggravated or the cases perpetuated self-mutilation or suicide, in which the patients were transferred to specialized psychiatric hospital with treatment in a closed environment. Patients’ respiratory rate, pulse rate, blood pressure, and heart rate were closely monitored during the treatment.

### 2.7 Outcome definitions evaluate

All personnel were repetitively trained for 3 days before study to be fully informed of the objective, requirement, methodology, and content of the study. The cases were evaluated by HAMD according to the following criteria. Reduction ratio = (total score before treatment − total score after treatment)/total score before treatment. Full recovery was considered if the reduction ratio ≥75% after 4 weeks of treatment; marked improvement was considered if reduction ratio was 50% to 74%; moderate improvement was considered if the reduction ratio was 26% to 49%; and ineffectiveness was considered if the reduction ratio ≤25%. Effective rate = the patients with reduction ratio ≥26%/total completed patients in group. Early improvement was considered if the reduction ratio was ≥20% after 2 weeks of treatment.

### 2.8 Statistical analysis

Data were collected on standard forms, checked for completeness, double keyed, and analyzed with SPSS 17.0 (SPSS, Inc., Chicago). Kolmogorov–Smirnov was applied for testing normality before analyses. Continuous variables were described as mean ± standard deviation. Frequencies and proportions were used for categorical variables. Two-way ANOVA with post hoc analysis was applied to detect the difference among different time points (within group) and between different groups. Chi-square test or *t* test were applied to analyze the response rate and demographics between groups. *P* < .05 (2-sides) was considered statistically significant in all analysis.

## 3 Results

Total of 134 patients were recruited, but only 103 subjects had completed all treatments. The mean age was 68.2 ± 9.37 years old, and 65.1% of the subjects were female. The patients were randomly assigned with equal number into 2 groups. Fourteen patients were withdrawn in active rTMS group, and 7 withdrawn in sham rTMS group, for various reasons including uncomfort with rTMS, failure to complete psychological assessment, severe physical illness, or no reason (Table 1). Finally, 48 patients finished the study in active rTMS group, and 55 patients finished the study in sham rTMS group. No statistical significance was found on the baseline socio-demographic, including age, gender, education, occupation, marital status and ethnics, and illness characteristics (Table 2).

**Table 1:**
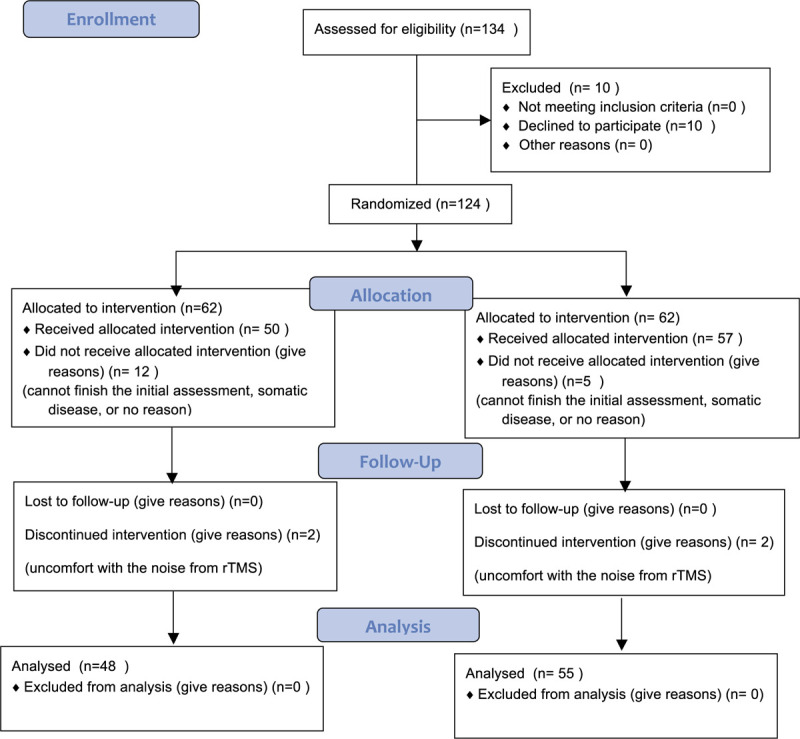
Details for patients grouping and dropping-out in 2 groups.

**Table 2:**
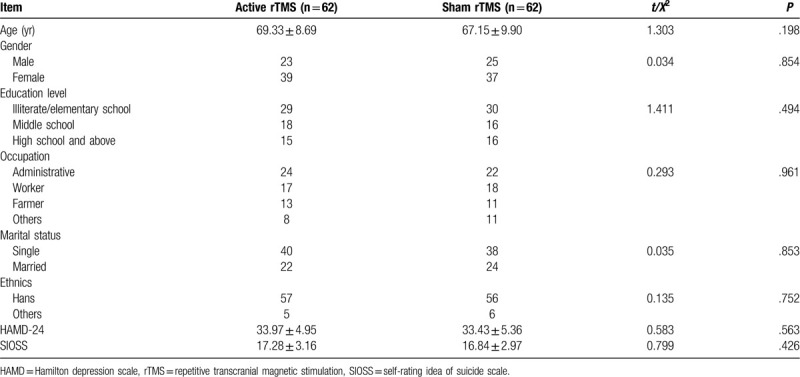
Comparison of baseline demographic and clinical characteristics between the patients in 2 groups.

The patients in both active and sham rTMS groups exhibited a substantial decrease in HAMD scores after 2 and 4 weeks of treatment. Further analysis revealed that the patients in in active rTMS group achieved a significant lower HAMD score, especially cognitive disorder and sleep disorder, when compared with sham rTMS group after 2 weeks treatment (*P* < .05). The improvement on HAMD score was significantly enhanced after 4 weeks of treatment in active rTMS group, when compared with that in sham rTMS group, including the factors of anxiety/somatization, cognitive disorder, retardation, and sleep disorder (*P* < .05) (Table 3). These results suggested that active rTMS, when compared to sham rTMS, exhibited substantial effect to reduce the clinical symptoms (HAMD score) in the elderly patients with depression.

**Table 3:**
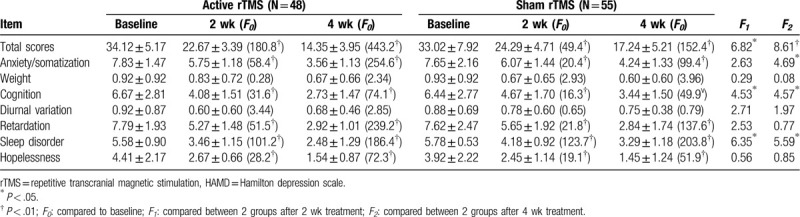
Reduction in 24-item Hamilton depression rating scale (HAMD-24) score over time in active rTMS versus Sham rTMS groups (mean ± SD).

All cases had a significant reduction in SIOSS scores after 2 and 4 weeks of treatment. As shown in Table 4, significant lower SIOSS score, especially sleep disorder and sense of despair, was noted in rTMS group when compared with sham rTMS group after 2 weeks treatment (*P* < .05) and 4 weeks treatment (*P* < .01). These results suggested that active rTMS, when compared to sham rTMS, exhibited substantial effect to reduce the suicidal ideation (SIOSS score) in the elderly patients with depression.

**Table 4:**
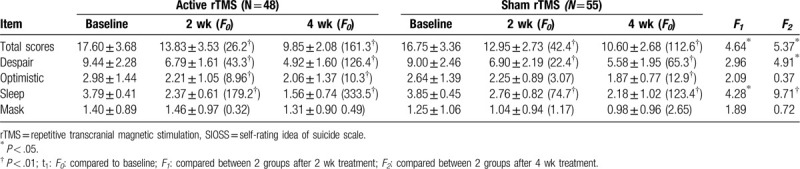
Reduction in self-rating idea of suicide scale (SIOSS) score over time in active rTMS versus sham rTMS groups (mean ± SD).

After 2 weeks of treatment, effective rate (HAMD reduction rate ≥26%) was significantly higher in rTMS group (25/48, 52.1%) versus sham rTMS group (18/55, 32.7%; *Χ*^*2*^ = 3.95, *P* < .05). Consistently, after 4 weeks of treatment, effective rate was significantly higher in rTMS group (45/48, 93.8%) versus sham rTMS group (46/55, 83.6%; *Χ*^*2*^ = 15.54, *P* < .01). These results suggested that active rTMS, when compared to sham TMS, exhibited a rapid onset and an enhanced effect to improve clinical symptoms in elderly patients with depression.

No serious adverse events occurred during the treatment. Five cases in rTMS group had dizziness, nausea, and chest tightness during the course of rTMS, in which 2 of them dropped out due to low tolerance and 3 cases achieved remission after symptomatic treatment. Three cases in the control group had nausea, mouth dryness, constipation, and headache during the treatment, and all of them achieved remission after symptomatic treatment. None suffered from epileptic seizures or other severe adverse events during treatment. No patients receiving active rTMS exhibited remarkable symptoms (or adverse effect) of abnormality of brain function. Mild headache was reported in early course of the treatment and rapidly decreased after repeated exposure in 4 patients receiving active rTMS.

## 4 Discussion

While the elderly population is constantly expanding in China, the suicide rate in the elderly people is 6 times higher compared with the younger people, in which 50% to 70% suicides occur secondary to depression. The probability of suicide in elderly patients with depression is 33.4 times higher compared with the ordinary population.^[17]^ Suicide is divided into completed suicide, suicide attempt, and suicidal ideation.^[18]^ Suicidal ideation is considered as the psychological activity that occurs at an early stage before completed suicide or suicide attempt. Hence, suicidal ideation is a risk factor for suicide and an effective intervention at this stage is important to prevent suicide.^[19,20]^ It has been reported that suicidal ideation is presented in 80.8% of the elderly people with depression versus 65.7% of the young and middle-aged people with depression.^[21]^ Drug therapy is currently the preferred option for reducing suicidal ideation, though it takes effect slowly. The present study found significant improvement in both groups (antidepressant therapy with active or sham rTMS), while the rTMS group exhibited more improvement on the clinical symptoms after 2 and 4 weeks of treatment. While antidepressant alone has effect to improve the clinical symptoms in depression patients,^[22]^ it is also of note to recognize the potential placebo effect of rTMS in various diseases including depression.^[23]^

Sleep disorder is the most common symptom in people with depression, which occurs particularly often in the elderly people with depression.^[24]^ The sleep quality decreases progressively with aging. Previous study showed that routine drug therapy combined with rTMS effectively improved the subjective sleep quality for the young and middle-aged people.^[25]^ The present study also reported that this combined therapy for 2 and 4 weeks was effective in improving the sleep quality for the elderly patients with depression. Meanwhile, it was further found that the all parameters in cognitive disorder, including self-guilt, suicide, agitation, depersonalization, derealization, paranoid, and obsession, were improved after 2 weeks of rTMS in elderly patients, and the improvement was more significant after 4 weeks of rTMS. Cognitive disorder, especially self-guilt, is critically associated with the risk of suicide in depression patients. The present study found that routine drug therapy combined with rTMS significantly improved the score in SIOSS assessment, which indicated a reduced suicidal ideation in the elderly patients with depression. While previous study reported that, in addition to improved sleep disorder and sense of despair, the sense of optimism was also improved in the young and middle-aged people with depression,^[21]^ an improvement of optimism was not observed in the elderly patients in the present study. This contradiction probably results from the insufficient social support and deterioration of physiological function in elderly patients. In the present study, the treatment, with or without rTMS, exhibited similar effect to improve anxiety, somatization symptoms, and retardation in the elderly patients.

In addition, routine drug therapy combined with rTMS exhibited therapeutic effect with quick onset for the elderly patients with depression. The marked effectiveness rate was 52.1% in the experimental group (vs 32.7% in the control group) after 2 weeks of treatment. This was consistent with the previous studies in the young and middle-aged patients (65.0% vs 38.8%).^[21]^ Another independent study also reported similar rapid effect of rTMS (57% vs 29%) in young and middle-aged patients with depression.^[26]^ In addition, the total effective rate of routine drug therapy with rTMS was significantly higher than that in control group (93.8% vs 83.6%), which was consistent with that in the young and middle-aged depression patients.^[27]^

While accumulating evidences demonstrated the application of rTMS in the treatment of neuropsychiatric disorders,^[8,9]^ the underlying mechanism is not adequately clear. Prevailing studies suggested that rTMS likely acted through the modulation of synaptic plasticity of neurocircuits in various brain regions, depending on the intensity, duration, and pattern of stimuli.^[28–30]^ Previous studies also indicated that application of rTMS might regulate the neurogenesis and neurotransmitter release in various brain regions, which contributed to its anti-depression activity.^[10]^ It was also noted that previous studies demonstrated the antidepression activity of rTMS in the patients with various ages. For instance, several multicenter randomized, controlled clinical trials reported remarkable improvement of the clinical symptoms in adolescent depression patients. Majority of these studies utilized the 10 Hz (ranging from 1 to 10 Hz) rTMS in left dorsolateral prefrontal cortex with 80% to 120% MT.^[31]^ The present study validated the therapeutic effectiveness of rTMS in the elderly patients, which potentially advanced the clinical treatment of this psychiatric disorder in elderly patients.

Several limitations existed in the present study. Active rTMS with frequency at 10 Hz and intensity of 100% MT was applied in the present study, then further studies with various frequencies (eg, 5 and 20 Hz) or intensities (eg, 80% or 120% MT) in a large scale of participants is needed to optimize the potential therapeutic effect of rTMS on the elderly patients. A few patients, for various reason, were dropped out from the study due to potential adverse issues for this treatment. Meanwhile, longitudinal follow-up is in need to investigate the long-term therapeutic effect of rTMS and recurrence rate of patients with rTMS treatment.^[23]^

## 5 Conclusion

Taken together, routine drug therapy combined with rTMS, when compared with drug therapy alone, demonstrated more rapid and significant effect to improve clinical symptoms, relieve depression, and reduce suicidal ideation in the elderly patients with depression. These results provided additional evidence to suggest the utilization of rTMS as an effective treatment in the elderly patients with depression. However, further studies are needed to optimize the parameters of rTMS, define the potential adverse effects, and determine the long-term therapeutic effect of rTMS in the psychiatric disorder.

## Author contributions

**Data analysis:** Lilei Dai and Peng Wang.

**Data collection:** Qingshan Guo, Hui Du, Fen Li, and Xinfu He.

**Manuscript preparation:** Lilei Dai, Peng Wang, and Rongrong Luan.

**Study integrity, concept, and design:** Lilei Dai, Rongrong Luan.

**Technical support:** Panpan Zhang.
